# 

**Published:** 1939

**Authors:** 


### The Medical Library of the University of Bristol

WITH WHICH IS INCORPORATED

**The Library of the Bristol Medico-Chirurgical Society.**

---

*The following donations have been received since the publication of the list in October,* 1939.

December, 1939.

| | |
|---|---|
| American Laryngological, Rhinological and Otological Society (1) .. .. .. | 1 volume. |
| Professor E. W. Hey Groves (2) .. .. | 3 volumes. |
| Milk Nutrition Committee (3) .. .. .. | 1 volume. |
| Professor C. Bruce Perry (4) .. .. .. | 3 volumes. |
| Baidya Nath C. Roy (5) .. .. .. .. | 1 volume. |
| Royal College of Surgeons (6) .. .. .. | 1 volume. |
| Bequest of Dr. J. E. Shaw (7) .. .. .. | 33 volumes. |
| Wilmer Ophthalmological Institute (8) .. | 1 volume. |

Unbound periodicals have also been received from Professor E. W. Hey Groves, Professor C. Bruce Perry, Dr. G. de M. Rudolf and Dr. J. Odery Symes.

### THE ONE HUNDRED AND SIXTY-NINTH LIST OF BOOKS.

The figures in round brackets refer to the figures after the names of the donors. The books to which no such figures have been attached have either been bought from the Library Fund or received through the *Journal*.

| | | | |
|---|---|---|---|
| | *Alcohol—Its Action on the Human Organism* .. .. .. | 3rd Ed. | 1938 |
| **Arber, A.** .. .. .. | *Herbals—Their Origin and Evolution.* | New Ed. | 1938 |
| **Beaumont, W.** .. .. | *Diathermy* .. .. .. .. .. .. .. .. .. | | 1939 |
| **Beaumont, W.** .. .. | *Infra-Red Irradiation* .. .. .. | 2nd Ed. | 1939 |
| **Chapman, H.**.. .. .. | *Thermae Redivivae: the City of Bath Described* .. .. .. .. .. .. .. .. | | 1673 |
| **Clay, H. H.** .. .. .. | *The Sanitary Inspector's Handbook* | 4th Ed. | 1939 |
| **Dogliotti, A. M.** .. .. | *Anesthesia* .. .. .. .. .. .. .. .. | (2) | 1939 |
| **Dunlop, D. M., and others (ed.).** | *Textbook of Medical Treatment* .. | | 1939 |
| **Edwards, H. C.** .. .. | *Diverticula and Diverticulitis of the Intestine* | | 1939 |
| **Fishberg, A. M.** .. .. | *Hypertension and Nephritis* .. | 4th Ed. | 1939 |
| **Gordon-Taylor, G.** .. | *The Abdominal Injuries of Warfare* | (2) | 1939 |
| **Ihre, B. J. E.** .. .. | *Human Gastric Secretion* .. .. .. .. .. | | 1939 |
| **Jordan, H. H.** .. .. | *Orthopedic Appliances* .. .. .. .. .. .. | | 1939 |
| **Jorpes, J. E.** .. .. .. | *Heparin* .. .. .. .. .. .. .. .. .. .. | | 1939 |
| **Katz, B.** .. .. .. | *Electric Excitation of Nerve* .. .. .. | | 1939 |

| | | |
|---|---|---|
| **Killian, H.** | *Pneumatopathien* (2) | 1939 |
| **Lister M.** | *De Fontibus Medicatis Angliae. Ed. alt. auct.* | 1684 |
| **Macdonald, G.** | *Reminiscences of a Specialist* | 1932 |
| *Medical Manual of Chemical Warfare* | | 1939 |
| **Meighan, C.** | *Treatise of Nature and Powers of the Baths of Bareges* | 1764 |
| **Miles, W. E.** | *Rectal Surgery* | 1939 |
| *Milk and Nutrition.* Part IV. | (3) | 1939 |
| **Oakes, L., and Bennett, A.** | *Materia Medica for Nurses* (4) | 1934 |
| **Olmsted, J. M. D.** | *Claude Bernard—Physiologist* | 1939 |
| **Osler, W.** | *Aequanimitas* 3rd Ed. | 1939 |
| **Porter, C., and Fenton, J.** | *Sanitary Law in Question and Answer* 4th Ed. | 1939 |
| **Power, S.** | *Surgical Diagnosis* | 1939 |
| **Robertson, W. G. A.** | *Aids to Forensic Medicine and Toxicology* 11th Ed. | 1939 |
| **Roy, B. N. C.** | *The Future of Medicine* (5) | 1939 |
| **Saunders, W.** | *A Treatise on Mineral Waters* 2nd Ed. | 1805 |
| **Troup, W. A.** | *Radiotherapy in Sinusitis* | 1939 |
| **Vernon, H. M.** | *Health in Relation to Occupation* | 1939 |
| **Wakeley, C. P. G. (ed.)** | *Modern Treatment in General Practice.* Vol. III. (4) | 1937 |
| **Warwick, F. J., and Tunstall, A. C.** | *First Aid to the Injured.* 17th Ed. | 1939 |
| **Wheeler, A., and Jack, W. R.** | *Handbook of Medicine* 9th Ed. (4) | 1934 |
| **Wilbur, R. L.** | *The March of Medicine* | 1938 |

### TRANSACTIONS, REPORTS, JOURNALS, ETC.

| | |
|---|---|
| Calendar of the Royal College of Surgeons, 1939 (6) | 1939 |
| Collected reprints of the Wilmer Ophthalmological Institute. Vol. V (8) | 1939 |
| Medical Directory, 1940 | 1939 |
| Mémoires de l'Académie Royale de Chirurgie, Tomes I–XV | 1743–1774 |
| Quarterly Cumulative Index Medicus. Vol. 25 | 1939 |
| Transactions of the 43rd Annual Meeting of the American Laryngological, Rhinological and Otological Society (1) | 1937 |
| Transactions of the Ophthalmological Society of the United Kingdom. Vol. LIX, Part 1 | 1939 |

### BEQUEST OF DR. J. E. SHAW (7.)

| | | |
|---|---|---|
| **Ader, Gullelmus** | *Enarrationes, de Ægrotis, et Morbis in Evangelio* | 1621 |
| **Albucasis** | *Octavii Horatiani.* | 1532 |
| **Anthonie, Francis** | *The Chemical Medicine or the Virtue of Potable Gold.* (No title page) | 1616 |
| **Barry, Ed., Kt.** | *Observations . . . on the Wines of the Ancients* | 1775 |
| **Bartholinus, Thoma** | *De Unicornu Observationes Novæ.* Ed. 2a | 1678 |
| **Bartholow, Roberts** | *On the Antagonism between Medicines* | 1881 |
| **Bauhini, Casparus** | *Anatomes. Liber Primus* | 1591 |
| **Blasius, Gerardus** | *Anatome Animalium* | 1681 |
| **Boord, Andrew** | *The Breviarie of Health* | 1587 |

| | | |
|---|---|---|
| **Bourneville and Regnard** | *Iconographie Photographique de la Salpêtrière.* 2 vols. | 1877–8 |
| **Bright, T.** | *Treatise of Melancholy* | 1586 |
| **Dispensary, The** | *A Poem in 6 Cantos.* Ed. 4. | 1700 |
| | *A Poem in 6 Cantos.* Ed. 9. | 1726 |
| **Graaf, R. de** | *De Mulierum Organis.* | 1672 |
| **Guidottus, T.** | *De Thermis Britannicis* | 1691 |
| **Guillemeau, J.** | *De l'Heureux Accouchement des Femmes* | 1609 |
| **Hall, J.** | *Select Observations on English Bodies. . . .* | 1679 |
| **Hartman, G.** | *The Family Physician.* | 1696 |
| **Hippocrates** | *Eight Sections of . . . Aphorismes* | 1665 |
| **Joubertus, L.** | *Paradoxarum* | 1565 |
| **Keith, A.** | *The Antiquity of Man* | 1920 |
| **Levens, P.** | *The Pathway to Health.* (No title page) | 1587 |
| **Lubbock, J., Kt.** | *Origin of Civilisation.* Ed. 2 | 1870 |
| **Markham, G.** | *Masterpiece.* (No title page) | 1688 |
| **Moreau, R.** | *Schola Salernitana* | 1625 |
| **Munk, W.** | *The Gold-headed Cane* | 1884 |
| | *The Gold-headed Cane.* Ed. 2 | 1828 |
| **Nixon, J. A., and Nixon, D. G. C.** | *Textbook of Nutrition* | 1938 |
| **Phioravant, L.** | *Three Exact Pieces of . . . .* | 1652 |
| **Salmon, W.** | *Complete English Physician* | 1693 |
| **Schoockius, M.** | *Disquisitio Physica* | 1659 |
| **Singer, C.** | *Short History of Medicine* | 1928 |
| **Stevens and Liebaut** | *De Landtwinninghe* | 1582 |
| **Tunstall, J.** | *The Bath Waters* | 1850 |
| **Turner, W.** | *The Names of Herbes* | 1881 |
| **Venner, T.** | *Via Recta ad Vitam Longam* | 1638 |
| **Willis, T.** | *The London Practice of Physick* | 1689 |
| **Woodall, J.** | *Surgeon's Mate.* (No title page) | 1639 |

### Publications Received

From MESSRS. BAILLIÈRE, TINDALL AND COX:

*The Psychological Aspect of Delinquency.* General Editor, G. DE M. RUDOLF, M.R.C.P., D.P.M., D.P.H. Price 2s. 6d.

From JOHN BALE MEDICAL PUBLICATIONS LTD.:

*Psycho-analysis.* By EDWARD GLOVER, M.D. Price 12s. 6d.

From CARDIFF MEDICAL SOCIETY:

*Proceedings of the Cardiff Medical Society, Session* 1938–39.

From MESSRS. H. K. LEWIS & CO. LTD.:

*A Text-book of X-ray Diagnosis by British Authors.* Vol. III. Edited by S. COCHRANE SHANKS, M.D., M.R.C.P., PETER KERLEY, M.D., and E. W. TWINING, M.R.C.S., M.R.C.P. Price £3 3s. 0d.

From MESSRS. E. & S. LIVINGSTONE ;

*Irving's Anatomy Mnemonics.* By ALASTAIR G. SMITH, L.R.C.P. and S. (Edin.). Fourth Edition. Price 1s. 6d.

*The Inometer or Ladder Food Chart.* By T. JOHNSON, D.Sc., F.L.S. Price 6d.

*Fractures and other Bone and Joint Injuries.* By R. WATSON-JONES, B.Sc., M.Ch.Orth., F.R.C.S. Price 50s.

FROM MESSRS. OLIVER & BOYD LTD. :

*The Essentials of Medical Treatment.* By DAVID MURRAY LYON, M.D., D.Sc., F.R.C.P. (Edin.). Price 15s.

From MESSRS. JOHN WRIGHT & SONS LTD. :

*Warwick and Tunstall's First Aid to the Injured and Sick.* Seventeenth edition. By F. C. NICHOLS, M.C., M.B., Ch.B., M.R.C.S., L.R.C.P., L.D.S. Price 2s. 6d.

*A Synopsis of Surgical Anatomy.* By ALEXANDER LEE MCGREGOR, M.Ch. (Edin.), F.R.C.S. (Eng.). 4th Edition. Price 17s. 6d.

*British Journal of Surgery.* Vol. XXVII, No. 106. Price 12s. 6d.

*Massage and Remedial Exercise.* By NOËL M. TIDY. 4th Edition. Price 15s.

*A Synopsis of Surgery.* By ERNEST W. HEY GROVES, M.S., M.D., B.Sc. (Lond.), F.R.C.S. (Eng.). Eleventh edition, fully revised. Price 17s. 6d.

*Demonstrations of Physical Signs in Clinical Surgery.* By HAMILTON BAILEY, F.R.C.S. (Eng.). 7th Edition, revised. Price 21s.

*An Atlas of the Commoner Skin Diseases.* By HENRY C. G. SEMON, M.A., D.M. (Oxon), F.R.C.P. (Lond.). 2nd Edition. Price 42s.

*An Index of Treatment.* By various writers. Edited by SIR ROBERT HUTCHISON, Bart., M.D., LL.D., P.R.C.P., assisted by REGINALD HILTON, M.A., M.D., F.R.C.P. 12th Edition, revised. Price 42s.

### Local Medical Notes

### APPOINTMENT.

Surgeon-Captain T. B. Dixon, V.D., R.N.V.R., to be Honorary Physician to H.M. the King.

### EXAMINATION RESULTS.

**University of Bristol**—Students of the University have recently passed the following examinations :—

M.D.—J. S. W. Little (by dissertation).

M.B., CH.B.—*Second Examination. Pass :* J. H. Challenger, Phyllis D. Moses, Leela Ready, I. F. R. Sutherland. *In Group II (Completing Examination) :* Jyotikana Datta,

Elizabeth Hodson. *In Group I only:* Moragh J. Dickson, Eileen M. Herbert, A. J. F. Saxton, Isabel M. M. Wilson.

M.B., CH.B.—*Final Examination.* (*Section I.*) *Pass:* R. G. Boyd (1, 2), N. F. W. Brueton, T. J. Butler (1, 2), Gwendolen M. Higgins, P. Jacobs (1, 3), Megan E. Jones, J. M. Joshua, J. K. Lewis, H. Mullen, J. W. J. Newton (1, 2), Marjorie Organ (1, 2, 3), R. W. Orton, R. D. R. Shanks, Patricia M. Simpson (1, 2), J. J. de S. Snijman, Barbara F. Thomas (1, 2), Joan Threadgold, G. H. Tovey, J. B. Tucker, D. N. Walder (1, 2), L. Willoughby (1, 2).

M.B., CH.B.—*Final Examination. Pass:* Ruth Appleby, D. C. Bodenham, Mary Crago, Marjorie Dainton, J. N. P. Davies, Sara M. Field-Richards, Joan M. Foss, E. M. Grace, Rosemary W. Knowles, J. E. Smith, A. A. Stonehill, Edith M. Wagstaff, Monica L. Hawkins, J. L. Elliott. *In Group I only:* Ruth M. Hunter.

B.D.S.—*Third Examination. Pass:* F. C. Bryant, P. B. Carey, G. K. G. Jennings, Sarah Jones, P. M. Nicholas (4), R. M. Sharp, Margaret J. Griffith.

B.D.S.—*Final Examination. Pass:* G. R. Styles.

L.D.S.—*Second Examination. Pass:* Isabella Rees, Leslie Murray.

L.D.S.—*Third Examination. Pass:* Jean M. Bryant, R. L. Hollington, V. T. Scard, G. J. Mills, R. S. Saxton, B. N. Neilson. *In Dental Materia Medica only:* L. Murray. *In Dental Mechanics only:* I. E. Perry.

L.D.S.—*Final Examination. Pass:* L. H. Barker-Tuft, J. C. Jowett, J. W. S. Jenkins. *Section I only:* D. G. Davies, J. R. Herbert, P. Le Masurier, O. H. Minton, C. R. Sage, R. B. Stiles.

**Royal College of Surgeons, Edinburgh.** — F.R.C.S. — Herbert L. Fuller.

**Conjoint Board.** — L.D.S. — *Pre-Medical Examination. Pass:* W. L. J. Crossman (Chemistry and Biology), J. B. Westlake (Physics), J. B. Wood (Biology).

(1) With Distinction in Materia Medica, Pharmacy and Pharmacotherapeutics.

(2) With Distinction in Toxicology.

(3) With Distinction in Pathology and Bacteriology.

(4) With Distinction in Dental Materia Medica.

L.R.C.P., M.R.C.S.—*First Examination. Pass :* Phyllis D. Moses (Anatomy and Physiology).

L.R.C.P., M.R.C.S.—*Final Examination. Pass :* Rotha H. Barnfield (5),* D. L. Bayley (6, 7), A. L. T. Beddoe (5),* D. C. Bodenham (5, 6, 7)*, Mary Crago (5, 6, 7),* J. N. P. Davies (5, 6, 7),* M. B. Dworkis (7),* J. M. Foss (5, 6, 7),* E. B. Hughes (5, 6, 7),* Megan E. Jones (6),* Joyce S. Miller (5, 6),* Mary R. More (5, 6),* D. Rosser (7), A. A. Stonehill (6),* E. W. J. Townsend (7), E. Meriel Wagstaff (5, 6, 7).*

* Qualified.

**Society of Apothecaries, London.** — L.M.S.S.A. — *Final Examination. Pass :* E. A. Griffiths (5, 8).

(5) Medicine. (6) Surgery. (7) Midwifery. (8) Forensic Medicine.

---

### Bristol Medico-Chirurgical Journal.

### NOTICE TO CONTRIBUTORS.

Illustrations should always be supplied ready for reproduction. All re-touching or other operations required will be charged to the Author. Line drawings should be drawn in Indian ink.

Authors of original articles are entitled to receive twenty-five Reprints, with covers showing source of Reprint free of charge. Additional Reprints may be obtained at the following rates (*provided notice is given at the time of returning proofs*) :—

### ADDITIONAL COPIES.

| | Copies | 8 pages | 16 pages |
|---|---|---|---|
| (*a*) With cover showing source of Reprint (no name or title) .. | 25 copies | 5/6 | 9/6 |
| | 50 „ | 8/6 | 16/- |
| (*b*) With special cover (showing source of Reprint, Author's name and title of paper) .. .. .. | 25 „ | 13/- | 17/- |
| | 50 „ | 18/- | 25/6 |

**Illustrations.**—For 25 copies of one page, **9d.** extra. For 50 copies of one page, **1/6** extra.

NOTE.—Contributors requiring special Covers on their free Reprints may obtain same at the following rates :—

Cover showing source of Reprint and Author's name and title of article .. .. .. } 25 copies, **7/6** 50 copies, **9/6**

### Index

Appendicitis, Acute.—H. Chitty, 241.
Appointments, 163, 222, 266.

Barrow Hospital, Opening of, 152.
Basal narcosis.—E. G. Bradbeer, 181.
Berry, R. J. A.—Investigation into the mental state of parents and sibs of 1,050 mentally defective persons, 189.
Book Reviews, 63, 141, 201, 252.
Bradbeer, E. G.—Basal narcosis, 181.
Burden, Mrs. R. G.—Obituary, 216.
Burden Neurological Institute, 154.
Burgess, N.—Seborrhœic dermatoses, 127.

Chesterman, C. C.—Forest folk: modern medicine in the Congo, 223.
Chitty, H.—Acute appendicitis, 241.
Colston Medical Research Fellowships, 76.
Colston Research Society, 155.
Congo, Modern medicine in the, 223.

Dermatoses, Seborrhœic.—N. Burgess, 127.
Diet in pregnancy.—J. A. Nixon, 165.
Drama, Medicine and the.—L. A. Moore, 1.

Examination Results, 76, 163, 222, 266.

Fells, A.—Obituary, 261.
Field Ambulance, The 144th, 211.
FitzGerald, C.—Obituary, 71.
Flemming, A. L.—Obituary, 212.
Forest Folk: modern medicine in the Congo, 223.

Library, 72, 158, 219, 263.
Long Fox Memorial Lecture: Forest folk: modern medicine in the Congo, 223.
Lovelock, J. E.—Some medical considerations of sports and games, 103.

Mayes, F. J. A.—Obituary, 215.
Medicine and the drama.—L. A. Moore, 1.
Mental defectives.—R. J. A. Berry, 189.
Moore, L. A.—Medicine and the drama, 1.
Myles, G. T.—Obituary, 261.

Narcosis, Basal.—E. G. Bradbeer, 181.
Nixon, J. A.—Diet in pregnancy, 165.

Obituaries—
Mrs. R. G. Burden, 216.
A. Fells, 261.
C. Fitzgerald, 71.
A. L. Flemming, 212.
F. J. A. Mayes, 215.
G. T. Myles, 261.
J. E. Shaw, 70.
M. F. Staniland, 262.

Obstetric emergencies, G. I. Strachan, 25.
Ogilvie, W. H.—Recent advances in surgery, 37.
Ophthalmology, Some recent advances in.—A. Palin, 85.

Palin, A.—Some recent advances in ophthalmology, 85.
Pregnancy, Diet in—J. A. Nixon, 165.
Presidential Address.—Medicine and the drama.—L. A. Moore, 1.
Prolapse, Prevention and Treatment.—H. J. D. Smythe, 177.
Publications received, 74, 162, 220, 265.

Rhinorrhœa, Spasmodic—E. Watson-Williams, 109.

Shaw, J. E.—Obituary, 70.
  Bequest of books, 264.

Short, A. R.—War surgery in Spain. 249.

Smythe, H. J. D.—Prevention and treatment of prolapse, 177.

Staniland, M. F.—Obituary, 262.

Society Meetings :—
  Bristol Medico-Chirurgical Society, 156, 217.
  B.M.A. (Bath, Bristol and Somerset Branch), 158.
  Galenicals, 218.

Spain, War surgery in.—A. R. Short, 249.

Sports and Games, Some medical considerations of.—J. E. Lovelock, 103.

Strachan, G. I.—Obstetric emergencies, 25.

Surgery, Recent advances in.—W. H. Ogilvie, 37.

War, The.—Editorial, 208.

War Surgery in Spain.—A. R. Short, 249.

Watson - Williams, E. — Spasmodic rhinorrhœa, 109.

